# *Candida albicans* in oral squamous cell carcinoma: a review of mechanistic pathways and clinical translational strategies

**DOI:** 10.3389/fcimb.2026.1923618

**Published:** 2026-09-28

**Authors:** Peining Zheng, Qin Xie, Kenneth K. Y. Cheng, Jordy Evan Sulaiman, Xiaojing Huang, Guowu Gan

**Affiliations:** 1 Fujian Key Laboratory of Oral Diseases & Fujian Provincial Engineering Research Center of Oral Biomaterial & Stomatological Key lab of Fujian College and University, School and Hospital of Stomatology, Fujian Medical University, Fuzhou, China; 2 Research Center of Oral Tissue Engineering & Institute of Oral Homeostasis, School and Hospital of Stomatology, Fujian Medical University, Fuzhou, China; 3 Department of Health Technology and Informatics, The Hong Kong Polytechnic University, Hung Hom, Kowloon, Hong Kong SAR, China; 4 Research Institute for Future Food (RiFood), The Hong Kong Polytechnic University, Hung Hom, Kowloon, Hong Kong SAR, China; 5 Research Centre for Chinese Medicine Innovation (RCMI), The Hong Kong Polytechnic University, Hong Kong, Hong Kong SAR, China; 6 Research Institute for Smart Ageing (RISA), The Hong Kong Polytechnic University, Hong Kong, Hong Kong SAR, China

**Keywords:** biofilm, *Candida albicans*, immune remodeling, oral mycobiome, oral potentially malignant disorders, oral squamous cell carcinoma, tumor microenvironment

## Abstract

Oral squamous cell carcinoma (OSCC) develops within a complex mucosal ecosystem in which microbial dysbiosis, epithelial injury, inflammation, and immune remodeling interact with established carcinogenic exposures. Although *Candida albicans* has repeatedly been associated with OSCC and oral potentially malignant disorders (OPMDs), its causal and translational significance remains uncertain. This review critically evaluates clinical, microbiological, mechanistic, and translational evidence, with particular attention to the distinction between association, biological plausibility, and causal inference. Current evidence is more consistent with context-dependent tumor-promoting activity than with independent carcinogenicity. Proposed mechanisms include acetaldehyde- and nitrosamine-related genotoxicity, inflammatory and immune-microenvironment remodeling, virulence factor-mediated epithelial injury, and biofilm-associated persistence; however, no single mechanism has been established as necessary or sufficient for *Candida*-associated tumor promotion. Longitudinal human evidence supports an association between increased fungal burden and malignant transformation in OPMDs, but no validated fungal-burden, virulence, or immune-cell threshold currently distinguishes colonization from pathological amplification. Candidate biomarkers, risk-stratification approaches, and fungal-directed interventions therefore remain investigational. Future studies should integrate standardized longitudinal sampling, spatially resolved fungal–bacterial–host analysis, and functional perturbation to determine whether *C. albicans* provides reproducible incremental value for clinical risk assessment or therapeutic targeting.

## 1 Introduction

According to GLOBOCAN 2024, an estimated 20.6 million new cancer cases and 9.8 million cancer deaths occurred worldwide across all cancers combined. Lip and oral cavity cancers accounted for approximately 452,000 new cases and 194,000 deaths globally (Sung et al., 2026). Oral squamous cell carcinoma (OSCC) is the predominant histopathological subtype, accounting for approximately 90% of oral malignancies (Ayuningtyas et al., 2022). Despite advances in multimodal treatment centered on surgery and combined with radiotherapy, chemotherapy, targeted therapy, or immunotherapy, patients with OSCC continue to face inadequate early detection, high rates of local recurrence, substantial treatment-related functional impairment, and limited improvements in long-term survival (Mohamad et al., 2023; Tan et al., 2023). Therefore, identifying additional contributors to OSCC initiation and progression, together with actionable targets, remains a major priority in current and future oral cancer research.

The oral microbiota is not a purely bacterial community, but a complex ecosystem comprising more than 700 bacterial species as well as fungi, viruses, archaea, and other microorganisms. This community may contribute to OSCC initiation and progression through chronic inflammation, microbial metabolites, immune modulation, epithelial barrier disruption, and remodeling of the tumor microenvironment. Previous OSCC microbiome research has focused predominantly on bacterial taxa, particularly *Fusobacterium nucleatum* (*F. nucleatum*) and *Porphyromonas gingivalis* (*P. gingivalis*), whereas the oral mycobiome remains comparatively less characterized (Sukmana et al., 2024).

*Candida* spp. are the yeasts most frequently isolated from the oral cavity and can colonize human mucosal surfaces, with carriage rates of up to 70% in healthy populations. Under homeostatic conditions, *Candida* spp. generally exist as commensals. However, when host defenses or local clearance mechanisms are impaired, or when local conditions favor colonization because of xerostomia, low salivary pH, immunosuppression, smoking, active caries, or denture wearing, *Candida* carriage and colonization burdens may increase and opportunistic infection may develop (Mun et al., 2016; Perić et al., 2024). Removable dentures can provide surfaces that favor *Candida* retention, adhesion, and biofilm formation, while *Candida*-associated denture stomatitis represents a common inflammatory condition among denture wearers (Perić et al., 2024). Clinical studies have reported increased *Candida* spp. detection or colonization burdens in patients with OSCC and oral potentially malignant disorders (OPMDs). Although *C. albicans* remains the most extensively studied *Candida* species in this context, non-albicans *Candida* species, including *C. krusei*, *C. tropicalis*, and *C. glabrata*, have also been identified in OSCC and OPMD cohorts, with their relative frequencies varying across studies (Roy et al., 2019; Sankari et al., 2020; Tasso et al., 2023). Accordingly, because mechanistic evidence is most developed for *C. albicans*, the remainder of this review focuses primarily on this species.

Two studies have further shown that OSCC-associated *C. albicans* isolates may exhibit enhanced biofilm formation, acetaldehyde production, and virulence-associated features (Alnuaimi et al., 2016; Subramani et al., 2024). These observations suggest that *C. albicans* may participate in OSCC-associated oral dysbiosis and may exhibit functional phenotypes relevant to tumor-associated microenvironmental changes and disease progression. However, the evidence so far does not support defining *C. albicans* as an independent, direct carcinogen in OSCC. A recent prospective cohort study likewise indicated that the association between *C. albicans* and malignant transformation of oral precancerous lesions may vary according to OPMD subtype and clinical context (Jiang et al., 2025).

On this basis, this review evaluates *C. albicans* as a context-dependent tumor-promoting factor rather than an independently sufficient carcinogenic driver. We examine the clinical and experimental evidence that distinguishes passive fungal detection or enrichment from functionally relevant tumor promotion, the host and lesion contexts in which such effects may occur, and the extent to which current evidence supports clinical risk assessment or intervention.

### 1.1 Literature search strategy

A literature search was conducted during manuscript preparation and updated during revision in September 2026. PubMed was used as the primary bibliographic database, with Google Scholar and supplementary academic search tools used for additional literature discovery. Search terms were selected according to the topics addressed in each section and included combinations of “*Candida albicans*,” “*Candida*,” “oral squamous cell carcinoma,” “OSCC,” “oral cancer,” “oral potentially malignant disorders,” “oral mycobiome,” “biofilm,” “candidalysin,” “*ECE1*,” “acetaldehyde,” “inflammation,” “immune microenvironment,” “epithelial–mesenchymal transition,” and “fungal–bacterial interaction.”

No formal publication-year restriction was applied. Recent studies were preferentially considered to reflect current developments, while older publications were retained when they provided foundational or primary evidence relevant to the topic. Studies were selected on the basis of their relevance to the clinical association between *Candida* and OSCC, proposed mechanistic pathways, or translational implications. Primary studies were preferentially consulted for specific mechanistic or experimental claims, whereas systematic and narrative reviews were used mainly to provide broader context and to identify additional relevant literature. Reference lists of key publications were also examined to identify additional studies. The literature was synthesized narratively, with emphasis on study design, experimental context, and the distinction between associative clinical evidence and mechanistic evidence.

## 2 Clinical association between *C. albicans* colonization and OSCC

### 2.1 Disease-associated enrichment of *Candida* spp. in OPMD and OSCC

Perera et al. used high-throughput sequencing to characterize the mycobiome of OSCC tissue biopsies and reported intralesional fungal dysbiosis characterized by reduced species diversity (Perera et al., 2017). By contrast, Berkovits et al. reported that both oral yeast carriage and fungal burden were significantly higher in patients with OSCC than in healthy controls; yeast burdens were also higher on tumor surfaces than on clinically healthy mucosa from the same patient, and a broader range of yeast genera could be cultured (Berkovits et al., 2016). Collectively, these findings suggest that OSCC-associated fungal dysbiosis may encompass reduced intratissue diversity, increased local yeast burden, and an altered spectrum of culturable yeasts on tumor surfaces. *Candida* spp. detection and colonization burdens have also been reported to be higher in oral samples from patients with OSCC than in healthy controls, supporting the presence of OSCC-associated fungal ecological alterations (Patil, 2020; Tasso et al., 2023). However, the specific fungal patterns reported across studies are not fully consistent, and their interpretation is influenced by differences in sampling and analytical methodology. A matched case-control study found that oral *Candida* spp. carriage and high colonization burden were significantly associated with increased oral cancer risk, even after adjustment for established risk factors such as smoking, alcohol consumption, and periodontal disease (Alnuaimi et al., 2015). Among positive samples, *C. albicans* is generally the predominant species. Its relative abundance is higher than that in healthy individuals, and some studies have reported that it accounts for up to 84% of OSCC samples positive for *Candida* spp. These observations provide the principal rationale for the emphasis on *C. albicans* in this review (Perera et al., 2017; Tasso et al., 2023).

The clinical significance of this enrichment extends beyond the superficial observation of increased *Candida* spp. detection or colonization burden; more importantly, it suggests that *Candida* spp. enrichment may occur across part of the OSCC disease continuum. Sankari et al. assessed *Candida* spp. in unstimulated saliva from 497 participants with OSCC, OPMD, or no oral disease and found carriage rates of 72.2%, 58.0%, and 20.5%, respectively. Mean colony burdens were also significantly higher in the OSCC and OPMD groups than in healthy controls (Sankari et al., 2020). These data indicate that *Candida* spp. enrichment is not restricted to established cancers but may also be present at the precancerous stage, consistent with its potential involvement in ecological changes across the OSCC continuum. A six-year retrospective cohort study of 48 patients with chronic hyperplastic candidiasis found epithelial dysplasia in 10 patients (20.83%) and malignant transformation in two patients (4.17%) (Zhang et al., 2021). Histopathological studies of oral leukoplakia have likewise indicated that *C. albicans*-positive lesions are more likely to exhibit epithelial dysplasia or other high-risk histological features, supporting consideration of *C. albicans* within frameworks for precancerous lesion risk assessment (Shukla et al., 2019). Further longitudinal evidence comes from a 2025 multicenter prospective cohort of 734 patients with OPMD recruited from seven hospitals in Taiwan. Chromogenic culture of lesion swabs showed that a high *C. albicans* burden was independently associated with malignant transformation, with the strongest association observed in patients with leukoplakia, indicating effect modification by OPMD subtype (Jiang et al., 2025).

The relationship between *Candida* spp. enrichment and the degree of malignant progression is not simply linear, and different oral niches may yield distinct fungal signals at the same disease stage (Heng et al., 2022). In a real-time polymerase chain reaction (PCR) analysis of formalin-fixed, paraffin-embedded oral biopsy specimens from nonsmokers, *C. albicans* and *C. tropicalis* were detected more frequently in mild-to-moderate epithelial dysplasia than in carcinoma *in situ* or OSCC, indicating that *Candida*-associated signals do not necessarily increase monotonically with histopathological progression (İlhan et al., 2023). Together with the longitudinal evidence described above, this finding underscores that *Candida*-associated signals may vary according to lesion stage, sampling niche, and study design. OSCC lesions commonly exhibit loss of mucosal integrity, tissue necrosis, and inflammation (Tan et al., 2023), all of which may create a niche favorable to *C. albicans* adhesion and proliferation. Thus, increased *C. albicans* abundance in OSCC may reflect active participation in tumor-associated microenvironmental remodeling, but it may also represent opportunistic colonization secondary to tissue alterations. Lesion-level enrichment should therefore be regarded as a clinical association signal rather than evidence of causality. The clinical evidence linking *Candida* spp./*C. albicans* with OPMD and OSCC is summarized in **Table 1**.

**Table 1:** Representative clinical evidence linking *Candida* spp. and *C. albicans* with OPMD and OSCC.

Study	Design and sample	Key finding
Alnuaimi et al. (2015)	Matched case-control; 52 oral cancer patients and 104 controls; oral rinse	Higher oral yeast carriage and *Candida* burden in cancer patients
Berkovits et al. (2016)	Cross-sectional; 20 OSCC patients and 40 controls; tumor and clinically healthy sites	Higher yeast carriage and burden, with a broader culturable yeast spectrum
Perera et al. (2017)	Tissue case-control; 25 OSCC tissues and 27 fibroepithelial polyps	Reduced fungal diversity and *C. albicans* enrichment in OSCC tissue
Sankari et al. (2020)	Cross-sectional; 497 participants with OSCC, OPMD, or no oral disease; saliva	Higher *Candida* carriage in OSCC and OPMD
Zhang et al. (2021)	Six-year retrospective cohort; 48 patients with chronic hyperplastic candidiasis	Epithelial dysplasia was present in 10/48 patients (20.83%), and malignant transformation occurred in 2/48 (4.17%).
Heng et al. (2022)	Cross-sectional multisite study across health, premalignancy, and carcinoma; saliva, plaque, and mucosa	Disease-associated microbial signals varied by oral niche
İlhan et al. (2023)	Retrospective FFPE study in nonsmokers; benign lesions, dysplasia, carcinoma *in situ*, and OSCC	*C. albicans* and *Candida tropicalis* were more frequent in mild-to-moderate dysplasia
Jiang et al. (2025)	Multicenter prospective cohort; 734 OPMD patients; lesion swabs	Higher baseline *C. albicans* burden was associated with malignant transformation, particularly in leukoplakia

FFPE, formalin-fixed, paraffin-embedded; OPMD, oral potentially malignant disorder; OSCC, oral squamous cell carcinoma.

### 2.2 Functional phenotypes of *C. albicans* isolates derived from OSCC

Compared with clinical enrichment data, the functional phenotypes of patient-derived isolates provide more direct insight into whether *C. albicans* could contribute to changes in the OSCC microenvironment. *C. albicans* isolates from different sources vary substantially in biofilm formation, metabolic activity, and virulence-associated functions. Isolates from patients with oral cancer have been reported to exhibit greater biofilm biomass and metabolic activity, a higher prevalence of high-acetaldehyde-producing phenotypes, and increased hydrolytic enzyme activity (Alnuaimi et al., 2016). Increased biofilm biomass and metabolic activity may promote long-term persistence on lesion surfaces and resistance to host clearance. Enhanced acetaldehyde production may increase epithelial exposure to metabolites capable of damaging DNA, whereas increased hydrolytic activity provides a biological basis for epithelial barrier disruption and tissue invasion. These phenotypes may also be particularly compatible with the tumor microenvironment. Marttila et al. found that, under hypoxic conditions with glucose as the carbon source, *C. albicans* isolates from patients with OSCC produced higher acetaldehyde levels, suggesting a potentially reinforcing interaction between tumor hypoxia, altered substrate availability, and fungal metabolism (Marttila et al., 2013). Compared with detection alone, these functional data more strongly suggest that OSCC-associated *C. albicans* may possess lesion-promoting potential.

Evidence at the virulence-gene level provides additional, but more limited, support for disease-associated fungal phenotypes. Candidalysin is a toxin peptide encoded by the extent of cell elongation 1 (*ECE1*) gene and is closely associated with *C. albicans* hyphal invasion, epithelial injury, and inflammatory activation (Engku Nasrullah Satiman et al., 2020). A PCR screening study of 60 clinical *C. albicans* isolates reported a higher prevalence of *ECE1* among isolates from patients with OSCC or OPMD than among those from healthy controls. However, detection of the *ECE1* gene does not establish its expression, candidalysin production, or lesion-level biological activity. These findings therefore support enrichment of a virulence-associated genetic feature in disease-derived isolates rather than establishing candidalysin as a clinical marker of malignant progression (Subramani et al., 2024). This distinction is further supported by recent experimental evidence showing no significant difference in tumor incidence between wild-type and candidalysin-deficient *ece1Δ/Δ C. albicans* in 4-nitroquinoline 1-oxide (4NQO)-conditioned mice, suggesting that candidalysin cannot currently be considered an indispensable determinant of the tumor-promoting phenotype (Wang et al., 2025). Together, these findings suggest that *ECE1*/candidalysin may contribute to epithelial injury and inflammatory signaling in specific contexts but should not be regarded as a standalone marker or necessary determinant of *Candida*-associated tumor promotion.

Altered functional phenotypes in patient-derived isolates do not necessarily imply that the isolates initiated tumor development. A more plausible interpretation is that the OSCC microenvironment selects *C. albicans* strains better adapted to local colonization, while high-virulence fungi may in turn exacerbate microenvironmental abnormalities, creating a bidirectional interaction.

### 2.3 Limitations of the clinical evidence: challenges in moving from association to causality

The clinical enrichment and isolate-level functional data summarized above support an association between *C. albicans* and OSCC, but the apparent magnitude and composition of this association vary substantially across studies. Reported *Candida* spp. prevalence and colonization burden may vary with host and clinical characteristics, including tobacco exposure, poor oral hygiene, xerostomia, immunosuppression or myelosuppression, and cancer treatment exposure (Grötz et al., 2003; Deng et al., 2010; Lalla et al., 2010; Bensadoun et al., 2011; Alnuaimi et al., 2015). Methodological heterogeneity provides an additional source of variation in reported fungal profiles, including differences in sampling compartment, fungal lysis and DNA extraction, internal transcribed spacer (ITS) target region, sequencing platform, taxonomic reference database, and downstream bioinformatic workflow. These factors limit direct cross-study comparability and are discussed in detail in Section 5.1.

Clinical context and treatment exposure may both influence *Candida* spp. colonization, particularly that of *C. albicans*. Patients with OSCC or other head and neck cancers commonly have systemic or local risk factors such as smoking, poor oral hygiene, denture use, xerostomia, antibiotic exposure, immunosuppression, or myelosuppression. Previous reviews have identified these factors as being associated with oropharyngeal candidiasis in patients receiving radiotherapy for head and neck cancer (Bensadoun et al., 2011). Elevated blood glucose in patients with diabetes may also induce hyphal morphogenesis in *C. albicans* and thereby facilitate disease progression (Martorano-Fernandes et al., 2020). Evidence for treatment-related disruption of the oral mycobiome is stronger. A systematic review found that oral fungal colonization increased from 48.2% before cancer therapy to 72.2% during treatment and remained at 70.1% after treatment; both head and neck radiotherapy and chemotherapy independently increased the risk of oral fungal infection (Lalla et al., 2010). Irradiation of the salivary glands markedly reduces salivary flow and compromises antimicrobial, anti-inflammatory, remineralizing, lubricating, and masticatory functions. In a prospective follow-up study, *Candida* spp. colonization peaked six months after radiotherapy, when salivary flow was lowest, and remained significantly above baseline at 12 months, indicating a persistent disruption of oral ecological homeostasis (Grötz et al., 2003). Clinical studies have also reported a significantly higher incidence of oral candidiasis during radiotherapy involving the oral cavity than during irradiation that spares the oral cavity (Deng et al., 2010).

Sampling compartment is particularly important because saliva or oral rinses, lesion-surface swabs, and tissue biopsies capture biologically distinct oral niches and should not be regarded as interchangeable (Aas et al., 2005; Dewhirst et al., 2010; Heng et al., 2022). Saliva and oral rinses integrate signals from multiple oral sites, whereas lesion swabs preferentially represent the lesion surface and tissue biopsies provide greater access to intralesional fungal signals. Differences in specimen type may therefore contribute substantially to variation in reported *C. albicans* detection and burden across studies.

Given these limitations, the available clinical evidence is more appropriately interpreted as associative rather than causal. Increased *C. albicans* abundance in OSCC or OPMD lesions may result from local barrier disruption, inflammatory exudation, treatment-related injury, or reduced salivary function; alternatively, it may further amplify existing pathological processes in specific host contexts and lesion niches. Current evidence therefore more strongly supports classifying *C. albicans* as a context-dependent tumor-promoting factor that may amplify OSCC-related processes in susceptible lesions, rather than as an independent carcinogenic driver.

## 3 Potential mechanisms by which *C. albicans* promotes OSCC progression

### 3.1 Metabolic tumor-promoting mechanisms: acetaldehyde, nitrosamines, and DNA damage

One important mechanism through which *C. albicans* may participate in oral carcinogenesis is the production or facilitation of carcinogenic metabolites capable of damaging DNA, particularly acetaldehyde and nitrosamines. Acetaldehyde has well-established genotoxic and mutagenic properties and is implicated in carcinogenesis. It reacts with DNA to form adducts and can induce intrastrand and interstrand crosslinks as well as single- and double-strand breaks, thereby increasing the risk of mutation and chromosomal instability (Thapa and Chan, 2025). Within the oral cavity, *C. albicans* can metabolize substrates such as ethanol or glucose to acetaldehyde. Marttila et al. showed that *C. albicans* produces substantial amounts of acetaldehyde from glucose under hypoxic conditions, suggesting considerable acetaldehyde-generating potential within the hypoxic tumor microenvironment (Marttila et al., 2013). This metabolic feature may interact with established OSCC risk factors. Smoking and alcohol consumption are classical risk factors for OSCC, and *C. albicans* acetaldehyde production may be further enhanced in these exposure settings. Gainza-Cirauqui et al. reported higher acetaldehyde production by isolates from smokers than by those from nonsmokers under the tested conditions, with the difference reaching statistical significance under ethanol–glucose incubation (Gainza‐Cirauqui et al., 2013). These findings suggest that tobacco exposure may influence candidal acetaldehyde metabolism, although the magnitude of this effect appears to depend on the experimental conditions. *Candida* spp. isolates from patients with oral cancer also commonly exhibit enhanced biofilm formation, increased biofilm metabolic activity, and high acetaldehyde production, indicating that robust biofilm growth and carcinogenic metabolic potential may coexist in OSCC-associated *C. albicans* (Alnuaimi et al., 2016). In addition to acetaldehyde, *C. albicans* may contribute to the formation of carcinogenic nitrosamines. A seminal study showed that, in the presence of nitrite and benzylmethylamine precursors, cultures of *C. albicans* generated more N-benzylmethylamine-derived nitrosamine (NBMA) than precursor-only controls, and NBMA is considered to have substantial carcinogenic potential. *C. albicans* may therefore provide another source of potentially DNA-damaging exposure by facilitating nitrosamine formation (Hsia et al., 1981). Concurrently, chronic mucosal inflammation induced by persistent *C. albicans* stimulation may intensify local oxidative and nitrosative stress. Studies of inflammation-associated carcinogenesis have shown that activated leukocytes, particularly neutrophils, generate reactive oxygen species (ROS) and reactive nitrogen species (RNS) that damage DNA bases. Unrepaired oxidative DNA damage can subsequently promote mutation and genomic instability (Mantovani et al., 2008; Dizdaroglu, 2012). *C. albicans* may thus contribute to OSCC-associated epithelial injury, genomic instability, and tumor progression by increasing exposure to genotoxic metabolites such as acetaldehyde and NBMA while superimposing ROS/RNS-mediated oxidative and nitrosative stress.

### 3.2 Inflammation and immune-microenvironment remodeling

Beyond metabolic injury, *C. albicans* may contribute to OSCC progression by inducing chronic inflammation and remodeling the immune microenvironment. Chronic inflammation is a well-established promoter of tumor development and can enhance tumor-cell proliferation, inhibit apoptosis, stimulate angiogenesis, facilitate immune evasion, and promote invasion and metastasis through activation of nuclear factor kappa B (NF-κB), signal transducer and activator of transcription 3 (STAT3), and mitogen-activated protein kinase (MAPK) signaling (Yu et al., 2009). *C. albicans* is sensed by host pattern-recognition receptors and induces multiple proinflammatory cytokines, thereby creating a persistent inflammatory background that may support abnormal epithelial proliferation and tumor progression. Studies of human OSCC tissues have begun to link *Candida* spp. colonization to host innate immune responses. Rusanen et al. assessed Toll-like receptors 1-10 (TLR1-TLR10), NF-κB, and *Candida* spp. colonization in newly diagnosed OSCC specimens and found increased TLR4 expression in the basement membrane region of samples positive for *Candida* spp., suggesting that *Candida* spp. colonization may be associated with altered TLR4-mediated innate immune sensing within OSCC lesions (Rusanen et al., 2024).

As indirect evidence that infection can induce systemic inflammation, early studies showed that systemic *C. albicans* infection increased serum tumor necrosis factor alpha (TNF-α) and interleukin 6 (IL-6) in mice in a time-dependent manner (Steinshamn and Waage, 1992). In mucosal infection models, *C. albicans* effectively activates epithelial NF-κB signaling and induces downstream inflammatory genes (Gratacap et al., 2013). These findings indicate that *C. albicans* not only colonizes mucosal surfaces but can also trigger inflammatory responses in epithelial and immune cells. Because sustained activation of the NF-κB/IL-6-STAT3 axis can promote proliferation, inhibit apoptosis, and establish a tumor-promoting microenvironment, chronic or recurrent *C. albicans* stimulation may provide an inflammatory context that favors abnormal epithelial proliferation and malignant transformation (G et al., 2025). The OSCC microenvironment itself is characterized by inflammatory-cell infiltration, epithelial barrier disruption, and dysregulated cytokine networks; *C. albicans*-induced inflammation may therefore compound the pre-existing inflammatory state of the tumor.

The potential link between *C. albicans* and immune evasion in OSCC has attracted increasing attention. Wang et al. showed that *C. albicans* upregulated programmed death-ligand 1 (PD-L1) in OSCC cells *in vitro* and in a mouse model, potentially through Toll-like receptor 2 (TLR2)/myeloid differentiation primary response 88 (MyD88) and TLR2/NF-κB signaling (Wang et al., 2022). By engaging programmed cell death protein 1 (PD-1) on T cells, PD-L1 suppresses antitumor immunity; *C. albicans* may therefore facilitate immune escape by enhancing PD-L1-mediated immunosuppression. Single-cell RNA sequencing has further shown that *C. albicans* infection remodels the immune-cell composition of OSCC. In a murine OSCC model, infection reduced the proportion of cluster of differentiation 8-positive (CD8^+^) T cells while increasing infiltration by interleukin 17A-positive (IL-17A^+^) cluster of differentiation 4-positive (CD4^+^) T cells, IL-17A^+^ γδ T cells, and myeloid-derived suppressor cells (MDSCs), particularly polymorphonuclear MDSCs (PMN-MDSCs). The increase in IL-17A^+^ γδ T cells correlated positively with tumor progression. Together, IL-17A^+^ cells and MDSCs can suppress CD8^+^ T-cell function and increase PD-1 expression, thereby contributing to an immune-evasive tumor microenvironment. These immune-cell shifts are mechanistically informative in murine oral-tumor models, particularly because neutralization of IL-17A or TCR γ/δ attenuated *Candida*-associated tumor progression (Wang et al., 2024). However, these murine immune alterations have not been validated as quantitative human thresholds; no established CD8:MDSC ratio, γδT17-cell percentage, or other immune-cell cutoff currently distinguishes passive colonization from pathological amplification.

Overall, available evidence suggests that *C. albicans* may sustain inflammatory signaling and promote immunosuppression within the OSCC microenvironment, thereby creating conditions favorable to tumor progression. However, a complete spatial and causal chain has not yet been demonstrated in human tissues. This mechanism should therefore be regarded as a biologically plausible tumor-promoting process rather than an established direct causal relationship.

### 3.3 Virulence factor-driven epithelial injury and invasive remodeling

The tumor-promoting effects of *C. albicans* may arise not only from microbial metabolites and inflammatory or immune modulation but also from its virulence factors. *C. albicans* can switch between yeast and hyphal morphologies, and hyphal formation enhances adhesion, invasion, and tissue damage. Virulence factors of particular relevance to OSCC include agglutinin-like sequence 3 (ALS3), candidalysin, and secreted aspartyl proteases (Saps).

ALS3 is an important hypha-associated surface adhesin that plays a central role in interactions between *C. albicans* and oral epithelial cells. Previous studies have shown that ALS3 mediates fungal adhesion to and invasion of host epithelial cells, whereas ALS3-deficient strains exhibit substantially reduced adhesion and invasion. These findings support an important role for ALS3 in oral epithelial adhesion and invasion, while its effects on epithelial damage and cytokine responses appear to be partly dependent on fungal burden and the broader infection context (Wächtler et al., 2011; Murciano et al., 2012). In the OSCC setting, sustained ALS3-mediated adhesion and invasion may prolong contact between fungal and tumor epithelial cells and create conditions conducive to virulence factor release, inflammatory stimulation, and barrier injury.

Candidalysin is a *C. albicans* peptide toxin that has received considerable attention in recent years. Encoded by *ECE1*, it links hyphal invasion to epithelial injury and inflammation. Candidalysin directly disrupts oral epithelial cell membranes and induces epithelial damage, activating signaling pathways involved in proliferation, inflammation, and tissue repair, including the epidermal growth factor receptor, p38 MAPK, and NF-κB pathways (Verma et al., 2017; Engku Nasrullah Satiman et al., 2020). Candidalysin can also activate the NOD-like receptor family pyrin domain containing 3 inflammasome in macrophages, promoting caspase-1-dependent release of interleukin 1 beta (IL-1β) and thereby providing a potential mechanism for inflammatory amplification (Kasper et al., 2018). Candidalysin can therefore couple epithelial injury with inflammatory signaling in mucosal infection models. However, these mechanistic activities do not establish candidalysin as a necessary determinant of *Candida*-associated tumor promotion.

The Sap family constitutes the major extracellular protease system of *C. albicans*, and Sap6 is of particular interest in oral mucosal infection. Kumar et al. showed that Sap6 binds host integrins through its arginine-glycine-aspartic acid motif, activates protease-activated receptor 2 (PAR2)-dependent p38 MAPK signaling, induces inflammatory mediator release, and promotes degradation of junctional proteins such as E-cadherin, thereby causing mucosal inflammation and compromising epithelial barrier integrity (Kumar et al., 2022). E-cadherin is a central determinant of epithelial adhesion and polarity, and its downregulation is a hallmark of epithelial-mesenchymal transition (EMT). PAR2 is also upregulated in OSCC and has been implicated in proinflammatory signaling, tumor-cell proliferation, and migration (Johnson et al., 2016b). Taken together, these findings provide a biologically plausible mechanistic link between Sap6-mediated epithelial injury and PAR2-associated signaling relevant to OSCC. However, a direct Sap6–PAR2 pathway driving OSCC progression has not yet been demonstrated (Kumar et al., 2022).

Persistent epithelial barrier disruption and inflammatory signaling may further drive invasive remodeling of OSCC cells, with EMT, matrix metalloproteinases (MMPs), and extracellular matrix degradation representing central downstream events. MMPs are not intrinsic virulence factors of *C. albicans*; rather, they are invasion-associated effectors produced by tumor or stromal cells in response to fungal stimulation. By degrading the extracellular matrix and basement membrane, MMPs facilitate tumor-cell migration, local invasion, and metastasis (Westermarck and Kähäri, 1999). Mechanistically, *C. albicans* may therefore indirectly activate MMP/EMT-associated invasion programs through virulence factor-mediated epithelial injury and inflammatory activation. This model is supported by *in vitro* and *in vivo* experiments. Vadovics et al. showed that *C. albicans* increased MMP activity in OSCC cells and increased the number of migratory tumor cells detaching from cell aggregates *in vitro*. In a xenograft model, OSCC tissues coinfected with *C. albicans* exhibited more pronounced inflammation and EMT, accompanied by decreased E-cadherin expression and increased vimentin and p63 expression (Vadovics et al., 2022). These findings are consistent with established associations between reduced E-cadherin expression and invasive or metastatic behavior in OSCC (Tanaka et al., 2003; Diniz-Freitas et al., 2006; Hashimoto et al., 2012; Lorenzo-Pouso et al., 2023). Junctional protein degradation, barrier injury, and inflammatory activation induced by *C. albicans* virulence factors may thus converge with EMT and matrix remodeling to create a proinvasive microenvironment.

### 3.4 Biofilms and context-dependent cross-kingdom interactions

Among the mechanisms through which *C. albicans* may participate in OSCC initiation and progression, biofilm formation is not an isolated event. Rather, biofilms provide a structural platform that localizes and sustains carcinogenic metabolism, chronic inflammation, immune evasion, virulence factor release, and epithelial barrier injury. Compared with planktonic cells, biofilm-associated *C. albicans* exhibits greater tolerance to antifungal agents, resistance to physical clearance, immune evasion, and local persistence (Lohse et al., 2018). The significance of biofilms therefore extends beyond fungal survival: they convert transient and dispersed stimulation of the oral mucosa or tumor surface into prolonged, localized microenvironmental pressure.

*C. albicans* forms highly organized biofilms on oral mucosal surfaces. These structures typically contain yeast-form and hyphal cells, extracellular matrix, co-colonizing bacteria, and host-cell components (Dongari-Bagtzoglou et al., 2009; Tsui et al., 2016). The biofilm extracellular matrix forms a dense physical barrier that limits antifungal penetration. Biofilm cells also undergo metabolic reprogramming, reduce overall metabolic activity, and upregulate genes encoding drug-efflux pumps, resulting in marked antifungal tolerance in biofilm-associated *C. albicans* (Lohse et al., 2018).

Mature *C. albicans* biofilms also resist innate immune clearance. Compared with early biofilms and planktonic cells, mature biofilms are less susceptible to neutrophil-mediated killing. This resistance does not result from neutrophil death or inactivation; rather, mature biofilms fail to effectively trigger the neutrophil ROS burst (Xie et al., 2012). Biofilm extracellular matrix also inhibits the formation of neutrophil extracellular traps (NETs), such that neutrophils may adhere to hyphae but remain unable to form effective net-like structures for capture and killing (Johnson et al., 2016a).

Within the oral ecosystem, *C. albicans* participates in cross-kingdom interactions with multiple bacterial species, but the biological consequences of these interactions are not uniformly synergistic. Interactions with oral bacteria may involve physical adhesion, signaling, metabolic exchange, or competition, and their effects depend on the microbial partners and experimental context (Du et al., 2022; Lueyar et al., 2023). Notably, Bor et al. demonstrated that direct contact between *C. albicans* and *F. nucleatum* inhibited *C. albicans* growth and hyphal morphogenesis, with inhibition of hyphal formation mediated by the *Candida* Flo9–fusobacterial RadD interaction. This interaction attenuated virulence-associated phenotypes rather than enhancing them, providing direct evidence that fungal–bacterial interactions can be antagonistic or mutually tempering (Bor et al., 2016). In contrast, Lee et al. observed higher salivary *C. albicans* abundance in patients with OSCC and positive abundance correlations with several bacterial taxa, including *T. denticola* and *L. casei* (Lee et al., 2024). However, this small saliva-based study demonstrates co-occurrence and correlation rather than direct physical interaction, spatial colocalization, or functional synergy within OSCC tissue.

Functional effects of polymicrobial biofilms on oral epithelial and cancer cells are likewise context dependent. Arzmi et al. showed that effluents from *C. albicans*, *Actinomyces naeslundii*, and *S. mutans* monospecies or polymicrobial biofilms differentially altered extracellular-matrix adhesion and inflammatory responses in malignant and non-malignant oral keratinocytes. IL-6 and IL-8 expression increased in malignant H357 cells after exposure to several biofilm effluents, whereas changes in E-cadherin and vimentin were not consistently indicative of epithelial–mesenchymal transition (Arzmi et al., 2019). Thus, these experiments support biologically relevant cross-kingdom modulation of oral cancer-cell phenotypes but do not establish a uniformly synergistic or pro-invasive effect. Separately, *Candida* spp. isolates from patients with OSCC have been reported to exhibit increased biofilm biomass, metabolic activity, and acetaldehyde production (Alnuaimi et al., 2016), indicating that persistent fungal colonization and tumor-relevant fungal phenotypes may coexist without demonstrating that bacterial partners necessarily enhance these effects.

Importantly, current human OSCC evidence has not yet established species-resolved spatial colocalization or direct functional interaction between *C. albicans* and specific bacterial partners within tumor tissue. Paired tumor–adjacent tissue studies have identified bacteriome–mycobiome alterations and cross-domain abundance correlations, but such analyses do not demonstrate physical interaction (Mukherjee et al., 2017). Spatial profiling has demonstrated bacterial microniches, including *Fusobacterium*-enriched regions, within human OSCC, but fungi were not profiled in the same spatial framework (Galeano Niño et al., 2022). Accordingly, co-detection or abundance correlation should be distinguished from spatial colocalization, direct physical interaction, functional synergy or antagonism, and causal contribution to tumor behavior.

In the context of OSCC, biofilms are therefore better regarded as platforms that sustain local microbial persistence and prolonged host exposure rather than as direct evidence of tumor promotion. Their biological consequences are likely to depend on fungal phenotype, bacterial partners, lesion context, and host responses.

Taken together, the mechanistic evidence reviewed in Section 3 supports a context-dependent model in which *C. albicans* is unlikely to act as an independently sufficient initiator of OSCC but may enhance tumor-relevant biological processes in susceptible, dysplastic, carcinogen-conditioned, or established tumor environments. Recent animal-model evidence is consistent with this interpretation. Prolonged *C. albicans* stimulation alone did not directly induce oral cancer in mice, whereas fungal exposure increased tumor formation in carcinogen-conditioned mucosa, supporting a tumor-promoting effect that depends on pre-existing context rather than an independent carcinogenic role (Wang et al., 2025). Importantly, no validated quantitative fungal-burden, virulence-gene, or immune-cell threshold currently exists that distinguishes passive colonization from pathological amplification. Accordingly, fungal burden alone, *ECE1* positivity, or a specific immune-cell ratio is not used here as a defining criterion. Instead, the term “pathological amplification” is used as an operational evidence concept: confidence in tumor-promoting activity increases when fungal enrichment is accompanied by measurable fungal activity, tumor-relevant host responses, and, ideally, temporal or perturbational evidence. This interpretation is testable rather than self-confirming. It would be weakened if prospective studies showed that fungal enrichment consistently followed rather than preceded the relevant disease progression, or if experimentally confirmed reduction of fungal activity or disruption of a proposed fungus-dependent pathway failed to attenuate the associated tumor-relevant phenotype. The context-dependent mechanisms by which *C. albicans* may promote OSCC progression are summarized in **Figure 1**.

**Figure 1:**
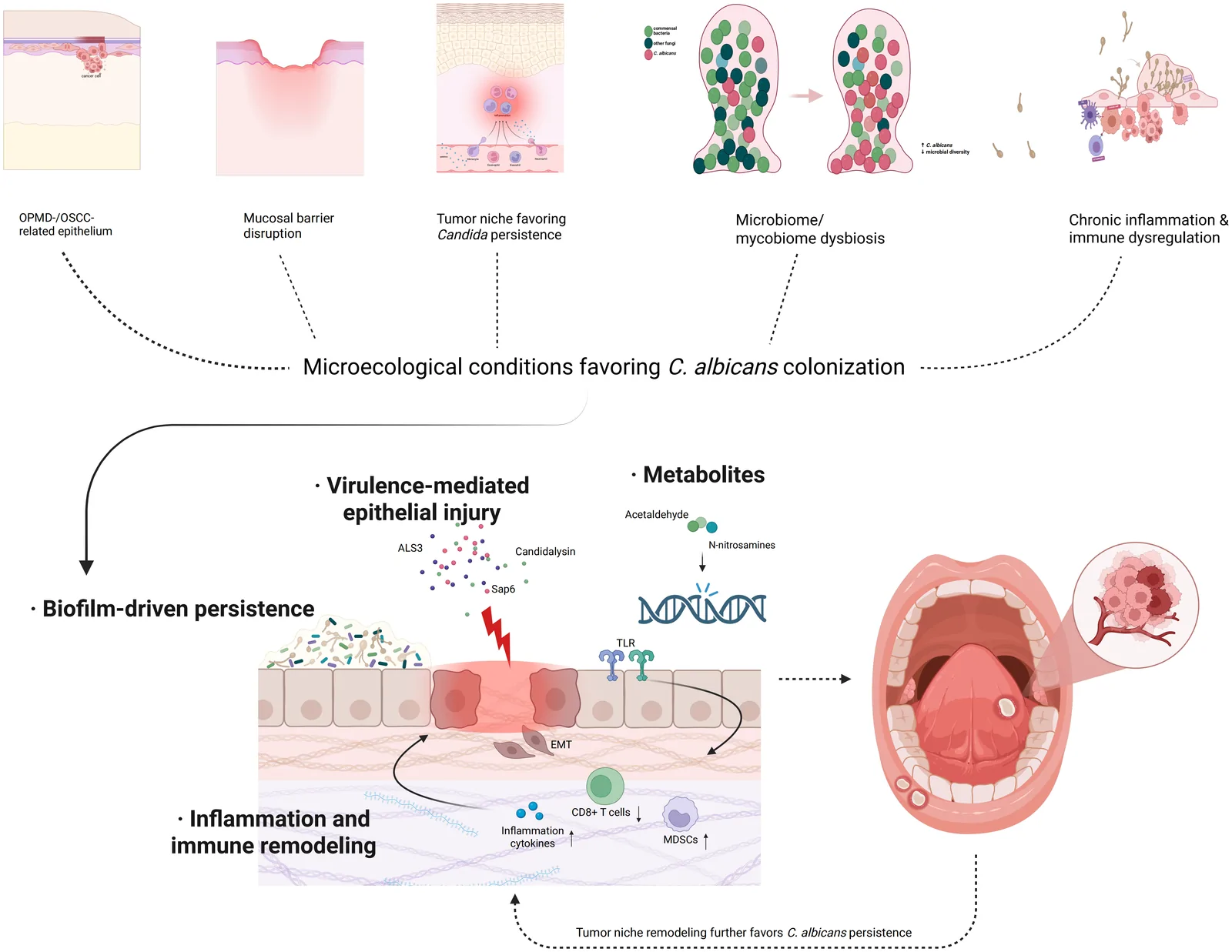
Context-dependent mechanisms by which *C. albicans* may promote OSCC progression. The schematic summarizes fungal persistence, metabolic genotoxicity, immune remodeling, epithelial injury, and biofilm-driven local stimulation within the dysplastic/OSCC microenvironment. Created in BioRender. Penny, Z. (2026) Available at: https://BioRender.com/2l38zop.

## 4 Clinical translational potential and intervention strategies

### 4.1 Potential diagnostic biomarkers

Current evidence supports considering *Candida*-related features as candidate research markers of OSCC or epithelial dysplasia, but is insufficient to regard them as validated clinical diagnostic biomarkers. Case-control and cross-sectional studies and systematic reviews have reported higher *Candida* detection rates, colony burdens, and virulence-related phenotypes in OSCC and some OPMDs (Ayuningtyas et al., 2022; Tasso et al., 2023; Wicaksono et al., 2025). However, existing studies have not shown that these measures provide stable and generalizable incremental diagnostic value beyond established clinical and histopathological assessment. Accordingly, this section addresses their potential translational value rather than established clinical tests.

#### 4.1.1 Salivary and fungal candidate markers

Saliva offers a minimally invasive means of quantifying oral fungal burden, although measurements may be influenced by host factors, lesion status, sampling site, collection procedures, and detection methods (Tasso et al., 2023; Tamai and Kiyoura, 2025; Wicaksono et al., 2025). Beyond *Candida* detection and colony burden, studies have reported *CaADH1* detection or expression in oral cancer-associated tissues (Hafed et al., 2019; Ayuningtyas et al., 2022), as well as greater biofilm biomass and metabolic activity, phospholipase and protease activities, and acetaldehyde production in isolates from patients with oral cancer (Alnuaimi et al., 2016; Wicaksono et al., 2025). These measures capture different aspects of fungal colonization and virulence; however, an elevated value alone cannot distinguish incidental or concomitant colonization from a fungal state that contributes to disease progression.

Notably, a 2025 multicenter prospective cohort study of patients with OPMDs defined >50 colony-forming units (CFU) on chromogenic culture as a high *C. albicans* burden and found this measure to be independently associated with malignant transformation (Jiang et al., 2025). However, the threshold was derived from the data distribution within that cohort and has not undergone independent external validation. Colonies were classified mainly by color and morphology on chromogenic culture without molecular confirmation at the species level. The study was designed to predict malignant transformation in OPMDs rather than to screen the general population for OSCC; therefore, its threshold should not be interpreted as a clinical diagnostic cutoff for OSCC. Fungal burden, virulence-gene expression, and composite *Candida* scores still lack external validation in independent prospective cohorts.

#### 4.1.2 Potential application of matrix metalloproteinase-9

Matrix metalloproteinase-9 (MMP-9) should be interpreted separately from *Candida*-specific candidate markers. Evidence from a systematic review identifies salivary MMP-9 as a candidate biomarker for OSCC and indicates that further research is required to validate its diagnostic value (Khijmatgar et al., 2024); the available evidence is insufficient to establish a universally applicable clinical cutoff. In addition, MMP-9 participates in extracellular matrix remodeling, inflammation, and tumor-related processes and is not specific to fungal exposure. Consequently, elevated MMP-9 cannot be used to infer the presence of *Candida* or to conclude that *Candida* is acting as a tumor-promoting amplifier.

Nevertheless, mechanistic studies provide a basis for investigating MMP activity in *Candida*-associated models. *In vitro* and mouse studies have shown that *C. albicans* exposure can increase total secreted MMP activity and alter the expression of several invasion-related MMP genes in OSCC models (Vadovics et al., 2022). These findings support a possible pathway-level link but do not establish diagnostic specificity. A panel combining fungal burden, virulence phenotypes, and MMP-9 remains hypothetical. Before clinical use, it should be evaluated against clinical and histopathological assessment and demonstrate incremental discriminatory value, adequate calibration, and external validity.

### 4.2 Risk stratification of oral potentially malignant disorders

Available studies show that *Candida*-related measures can differ between patient groups and healthy controls, but between-group differences do not directly translate into individual prediction of malignant progression in OPMDs. A systematic review and meta-analysis of six studies found oral *Candida* detection more frequent in patients with OSCC than in healthy controls, with a pooled OR of 9.50; *Candida* isolates from patients with oral cancer also showed greater biofilm formation, metabolic activity, phospholipase and protease activities, and acetaldehyde-producing capacity (Tasso et al., 2023). A corresponding case-control study likewise observed enhanced virulence phenotypes and acetaldehyde production among OSCC-associated isolates (Alnuaimi et al., 2016). However, these studies primarily compared patients with established OSCC with controls. They support disease-associated differences but cannot determine whether the measures precede malignant transformation or predict future progression of OPMDs. Experimental studies further show that *C. albicans* can promote tumor growth, invasion, and MMP-related changes in OSCC models, providing biological plausibility for its role as an amplifier of disease progression (Vadovics et al., 2022). Mechanistic evidence, however, cannot substitute for clinical prediction validation. The central question is therefore not whether fungi coexist with OSCC, but whether fungal burden, virulence phenotypes, or host-response markers consistently predict malignant transformation of OPMDs beyond established clinicopathological factors such as lesion type and degree of epithelial dysplasia.

#### 4.2.1 Available evidence and its boundaries

Evidence levels differ substantially across candidate markers. With respect to host-response markers, an exploratory study used single-cell transcriptomics and immunohistochemistry to compare normal oral mucosa, *C. albicans*-positive OPMD, *C. albicans*-negative OSCC, and *C. albicans*-positive OSCC tissues and observed the strongest stratifin (SFN) immunohistochemical staining in the *C. albicans*-positive OSCC specimen. However, each of the four groups comprised only one tissue specimen from one patient. Although 18,433 cells were analyzed, the number of cells does not replace biological replication at the patient level. The finding therefore only suggests that SFN may reflect a *Candida*-associated state of the epithelium or tumor microenvironment; it cannot be used to estimate diagnostic performance or predict malignant transformation (Hsieh et al., 2022).

Among fungal measures, fungal burden is currently supported by longitudinal association data from a multicenter prospective cohort (Jiang et al., 2025), but neither the external reproducibility of its candidate cutoff nor its incremental predictive value over existing clinicopathological models has been established. By contrast, evidence for *CaADH1* detection or expression derives mainly from a cross-sectional primary study and its systematic review (Hafed et al., 2019; Ayuningtyas et al., 2022), whereas biofilm formation, hydrolytic enzyme activity, and acetaldehyde production are supported primarily by case-control isolate studies (Alnuaimi et al., 2016). These findings provide biological plausibility but do not demonstrate prediction of malignant transformation in OPMDs. A recent scoping review also identified substantial heterogeneity in sampling methods, culture conditions, molecular identification techniques, study populations, and histopathological classifications. Because much of the evidence is cross-sectional or *in vitro*, it remains unclear whether fungal alterations are a cause or consequence of lesion progression (Wicaksono et al., 2025).

#### 4.2.2 Proposed validation strategy

To translate the context-dependent amplifier model into a testable research framework, future studies should use multicenter prospective OPMD cohorts with prespecified outcomes, including histological progression and malignant transformation. Given the current evidence hierarchy, priority should be given to testing the external reproducibility of fungal burden and its longitudinal changes, while treating SFN, *CaADH1*, and other molecular or virulence phenotypes as exploratory adjunct variables. At fixed follow-up intervals, standardized samples should be obtained from the same lesion site and paired non-lesional mucosa. Temporal trajectories of fungal burden, consistency of species or strain identity, virulence phenotypes, and host responses should be integrated to evaluate the relationship between persistent fungal states and lesion progression. Antifungal and antibiotic use, denture use, oral hygiene, and relevant exposures should also be recorded to reduce confounding. The Taiwanese cohort indicates that subsequent studies should not merely reproduce the >50-CFU cutoff; they should also test whether the predictive effect of fungal burden differs across OPMD subtypes and whether lesion-specific burden or longitudinal burden trajectories improve risk prediction. Because that study used a single baseline measurement, lacked fungal-burden data for approximately 25% of participants, and had limited precision in subtype analyses, the >50-CFU value should be treated as a study-specific prognostic cut point requiring external validation rather than as a biological threshold defining pathological amplification (Jiang et al., 2025). Candidate thresholds and combined markers should be validated in independent cohorts, and models should be tested for improvements in discrimination, calibration, or clinical net benefit beyond established clinicopathological factors such as lesion type and degree of epithelial dysplasia. Until such validation is available, neither these markers nor their combined models should be used for individual clinical risk stratification.

### 4.3 Emerging intervention strategies

#### 4.3.1 Preclinical investigation of antifungal drugs and fungal signaling molecules

Antifungal drugs and fungal-derived signaling molecules should be discussed separately according to the level of evidence. Farnesol is a quorum-sensing molecule produced by *C. albicans* and contributes to regulation of the yeast-to-hypha transition (Yang et al., 2023). An *in vitro* study using the OSCC9 and OSCC25 cell lines found that treatment with 30–60 μM synthetic farnesol for 48 h inhibited proliferation and enhanced apoptotic signaling. The authors stated that the findings were consistent across both cell lines, although the article primarily presented data from OSCC9 cells (Scheper et al., 2008). This evidence should therefore be regarded as deriving from a single *in vitro* study and does not establish *in vivo* efficacy, intratumoral exposure, therapeutic selectivity, or safety.

Itraconazole showed concentration- and time-dependent antiproliferative effects in four OSCC cell lines and inhibited tumor growth in patient-derived OSCC xenografts through downregulation of Hedgehog signaling (Ban et al., 2020). However, the study did not establish whether standard antifungal dosing can achieve salivary or OSCC tissue concentrations sufficient to reproduce these antitumor effects, nor whether antifungal and antitumor effects can be achieved simultaneously in patients. The official prescribing information indicates that systemic itraconazole exposure is formulation-dependent and warns of serious hepatotoxicity, congestive heart failure or other cardiac effects, and clinically significant cytochrome P450 3A4-mediated drug interactions (DailyMed, 2026). Itraconazole should therefore currently be described as a drug-repurposing candidate with preclinical evidence in OSCC rather than an established adjunctive treatment. Prospective studies are needed to evaluate pharmacokinetics within human lesions, interactions with antineoplastic therapy, and safety.

#### 4.3.2 Biofilm-targeted approaches

Biofilm-targeted strategies remain experimental. Studies of *C. albicans* biofilms indicate that adhesion contributes to fungal colonization and persistence; extracellular matrix, drug efflux pumps, and persister cells are associated with antifungal tolerance; and hydrolytic enzymes such as secreted aspartyl proteinases (Saps) primarily contribute to adhesion, tissue damage, and invasion (Dongari-Bagtzoglou et al., 2009; Tsui et al., 2016; Lohse et al., 2018). An *in vitro* study of clinical *C. albicans* and non-albicans *Candida* isolates found that 24 h of treatment with 100 ppm mycogenic silver nanoparticles reduced biofilm metabolic activity by more than 50%; inhibition of secreted aspartyl proteinase activity was also observed at 100 ppm (Hamid et al., 2018). However, these findings were obtained with a specific silver-nanoparticle preparation and an *in vitro* model. They do not show that the preparation penetrates the extracellular matrix of mature biofilms and provide no evidence on retention or distribution within OSCC lesions, oral mucosal safety, or clinical efficacy.

Low-level laser irradiation has likewise been evaluated only experimentally. In one study without a photosensitizer, *C. albicans* cultures were irradiated at 685 or 830 nm, after which mice were infected with the irradiated isolates. At energies of >10 J, irradiation inhibited *C. albicans* growth and increased survival in mice infected with the irradiated isolates, indicating reduced fungal pathogenicity (Seyedmousavi et al., 2014). The study did not deliver laser treatment directly to infected animals or OSCC lesions and did not address human oral candidiasis or mature tumor-associated biofilms. Findings obtained with this particular device and irradiation protocol cannot be directly extrapolated to clinical treatment. Silver nanoparticles and low-level laser irradiation should therefore be considered proof-of-concept strategies. Before clinical application can be considered, subsequent studies should directly quantify spatial delivery, matrix penetration, local exposure, mucosal toxicity, and biofilm clearance in human specimens.

#### 4.3.3 Experimental strategies involving immune checkpoints and microbiota modulation

Experimental studies have demonstrated measurable immunomodulatory effects of *C. albicans*. Both viable and heat-killed *C. albicans* upregulated PD-L1 expression in Cal27 and HN6 cells, with TLR2/MyD88 and TLR2/NF-κB signaling potentially contributing to this response; increased PD-L1 expression was also observed in the oral mucosal epithelium of normal and OSCC-bearing mice (Wang et al., 2022). In another study of oral tumor-bearing mice, single-cell RNA sequencing of 35,943 high-quality cells from tumor samples showed that *C. albicans* exposure was associated with fewer CD8^+^ T cells, increased IL-17A^+^ CD4^+^ and γδ T cells, and greater MDSC infiltration. Neutralization of IL-17A or TCRγ/δ attenuated *Candida*-associated tumor progression, whereas C-C motif chemokine ligand 2 (CCL2) neutralization reduced MDSC infiltration. *C. albicans* overgrowth also impaired the efficacy of PD-1 blockade (Wang et al., 2024). However, the study did not test whether antifungal treatment could restore the response to PD-1 blockade. Combining antifungal therapy with immune checkpoint inhibition therefore remains a testable hypothesis rather than an established treatment strategy.

An animal experiment provides relatively direct evidence that probiotic administration may modify oral carcinogenesis in a rat model. In a 4NQO-induced rat oral cancer model, continuous administration of high-dose *Lactobacillus salivarius* REN (5 × 10^10^ CFU/kg/day) from an early stage of carcinogenesis reduced the incidence of histologically confirmed papilloma or squamous cell carcinoma from 65% (13/20) in the 4NQO-only group to 0% (0/15), with a dose-dependent decline. The effect was accompanied by reduced oxidative DNA damage, lower cyclooxygenase-2 (COX-2) and proliferating cell nuclear antigen (PCNA) expression, and increased apoptosis. In addition, an *in vitro* co-incubation experiment in the same study showed that the strain promoted conversion of 4NQO-related carcinogenic compounds into less toxic metabolites. The marked effect may therefore partly reflect direct interference with this specific chemical carcinogenesis process and should not be equated with treatment of established human OSCC (Zhang et al., 2013). The evidence status and translational boundaries of these candidate strategies are summarized in **Table 2**.

**Table 2:** Evidence status, observed effects, and translational boundaries of candidate OSCC-related interventions.

Strategy	Model/exposure	Main signal	Evidence status and principal gap	References
Farnesol	OSCC9 and OSCC25 cells; 30–60 μM for 48 h	Reduced proliferation and enhanced apoptotic signaling	Single *in vitro* study; *in vivo* exposure, selectivity, and safety remain unknown	(Scheper et al., 2008)
Repurposed itraconazole	Four OSCC cell lines and patient-derived xenografts	Hedgehog inhibition with reduced proliferation and xenograft growth	Preclinical only; human-lesion pharmacokinetics and clinical efficacy remain unknown	(Ban et al., 2020; DailyMed, 2026)
Mycogenic silver nanoparticles	Clinical *Candida* isolates; 100 ppm for 24 h	Biofilm metabolic activity decreased by >50%; Sap activity was inhibited	*In vitro* only; mature-biofilm penetration, OSCC delivery, and safety were not assessed	(Hamid et al., 2018)
Low-level laser irradiation	*C. albicans* cultures irradiated at 685 or 830 nm; mice subsequently infected with irradiated isolates	At >10 J, irradiation inhibited *C. albicans* growth and increased survival in mice infected with irradiated isolates, indicating reduced pathogenicity	Laser was not delivered to infected animals or OSCC lesions; evidence is device- and protocol-specific	(Seyedmousavi et al., 2014)
Proposed antifungal therapy plus PD-1 blockade	*C. albicans*-exposed OSCC cells and oral tumor-bearing mice	PD-L1 upregulation, immune-cell remodeling, and reduced efficacy of PD-1 blockade	Mechanistic rationale only; antifungal rescue of the PD-1 response has not been tested	(Wang et al., 2022, 2024)
*Lactobacillus salivarius* REN	4NQO-induced rat model; 5 × 10¹^0^ CFU/kg/day	Neoplastic lesions decreased from 65% to 0%, with reduced DNA damage and proliferation markers	Chemoprevention model; the effect may partly reflect altered 4NQO metabolism and does not establish efficacy in human OSCC	(Zhang et al., 2013)

All listed strategies remain preclinical or investigational; none has established efficacy in *Candida*-associated OSCC.

4NQO, 4-nitroquinoline 1-oxide; CFU, colony-forming unit; OSCC, oral squamous cell carcinoma; PD-1, programmed cell death protein 1; PD-L1, programmed death-ligand 1; Sap, secreted aspartyl protease.

## 5 Limitations and future directions

### 5.1 Major limitations of the current evidence

The Taiwanese multicenter prospective OPMD cohort discussed above showed that the study-defined high baseline *C. albicans* burden was associated with subsequent malignant transformation (Jiang et al., 2025). This study advanced the evidence from cross-sectional association to an observational association with temporal ordering, but it did not establish causality. Fungal burden was measured only once at baseline; the >50-CFU threshold was derived from the cohort’s own data distribution; species assignments were not confirmed molecularly; strain-level variation and exposure to antifungal or antibacterial agents during follow-up were incompletely assessed; and residual confounding could not be excluded. The finding should therefore be interpreted as a prognostic association within a particular clinical context rather than a universal biological boundary.

Mechanistic studies have demonstrated measurable effects of *C. albicans* on OSCC cell signaling, tumor-associated immune-cell populations, and tumor growth in experimental systems (Wang et al., 2022, 2024, 2025). Human tissue studies have also identified OSCC-associated fungal profiles (Perera et al., 2017), while a prospective cohort found an association between lesion *Candida* burden and malignant transformation (Jiang et al., 2025). These forms of evidence address different questions: cell and animal models support biological plausibility, whereas human observational studies support association and potential prognostic value. No clinical intervention study has yet shown that reducing *Candida* burden prevents malignant transformation of OPMDs, improves response to OSCC treatment, or prolongs survival. Clinical translation therefore requires intervention studies with prespecified mycological and oncological endpoints and cannot be inferred solely from antifungal activity or preclinical antitumor effects.

Existing oral mycobiome studies differ substantially in sampling compartment and analytical workflow. Ghannoum et al. analyzed oral rinse samples from healthy participants using multitag 454 pyrosequencing of the ITS region (Ghannoum et al., 2010); Perera et al. analyzed deep OSCC tissues using ITS2/MiSeq (Perera et al., 2017); and Heng et al. compared three oral sites using ITS1/NovaSeq (Heng et al., 2022). Because sampling, amplified region, sequencing platform, and taxonomic workflow differ simultaneously, between-study differences in community composition or diversity may reflect biological variation, measurement processes, or both. These methodological differences and their interpretive boundaries are summarized in **Table 3**.

**Table 3:** Methodological differences and interpretive boundaries in oral mycobiome research.

Methodological domain	Representative differences	Interpretive boundary and minimum requirements
Sampling compartment	Oral rinse (Ghannoum); deep OSCC tissue (Perera); buccal mucosa, plaque, and saliva (Heng) (Ghannoum et al., 2010; Perera et al., 2017; Heng et al., 2022)	Oral compartments are not interchangeable, and relative abundance does not represent absolute burden. Report the exact site, specimen type, sampling time, and an absolute-quantification method when feasible.
DNA extraction and contamination control	FastPrep mechanical disruption; non-optimized tissue extraction; kit-based extraction without reported mechanical lysis; bead-beating evidence showing taxon-specific recovery (Ghannoum et al., 2010; Dupuy et al., 2014; Perera et al., 2017; Diaz et al., 2017; Heng et al., 2022)	No extraction method is uniformly unbiased. Report bead- and enzymatic-lysis parameters, blanks, mock-community recovery, batch layout, and decontamination rules.
Amplicon sequencing and taxonomic workflow	ITS1/454/AFTOL-NCBI; ITS2/MiSeq/UNITE; ITS1/NovaSeq/UNITE; non-oral multi-primer benchmarking and UNITE curation guidance (Ghannoum et al., 2010; Tedersoo et al., 2015; Perera et al., 2017; Nilsson et al., 2019; Heng et al., 2022)	Primer, read-processing, database, and classifier differences affect taxonomic resolution and assignment. Report primers, platform, processing parameters, database version, and confidence criteria.
Fungal burden measurement and threshold validation	Culture-based CFU in a prospective OPMD cohort; host-DNA-normalized ITS2 qPCR in OSCC tissue; relative amplicon profiles across oral sites (Perera et al., 2017; Heng et al., 2022; Jiang et al., 2025)	Culture CFU, host-normalized qPCR, and relative amplicon profiles are not interchangeable. Report detection limits and normalization procedures, and validate candidate thresholds in independent populations.

This table compares analytical workflows and their interpretive boundaries; it is not intended to classify any individual study result as a technical artifact.

AFTOL, Assembling the Fungal Tree of Life; CFU, colony-forming unit; ITS, internal transcribed spacer; NCBI, National Center for Biotechnology Information; OPMD, oral potentially malignant disorder; OSCC, oral squamous cell carcinoma; qPCR, quantitative polymerase chain reaction.

Methodological studies show that these differences can influence results. Dupuy et al. used an intensified bead-beating protocol and detected *Malassezia* (Dupuy et al., 2014). In standardized *Malassezia* suspensions, Diaz et al. compared bead-beating speeds of 5 and 6 m/s; the higher speed nearly doubled DNA recovery for *M. restricta* and *M. globosa* but did not affect recovery of *M. sympodialis* (Diaz et al., 2017). A multiple-primer benchmarking study in non-oral samples showed that primer combinations could substantially affect community profiles, although its effect estimates cannot be directly transferred to oral samples (Tedersoo et al., 2015). Ghannoum used sequence matching against AFTOL/WASABI and NCBI/GenBank (Ghannoum et al., 2010), whereas Perera and Heng used the UNITE database (Perera et al., 2017; Heng et al., 2022); these workflows are not fully equivalent in reference-sequence curation or taxonomic classification rules (Nilsson et al., 2019). Future studies should therefore report sampling, lysis, amplification, sequencing, controls, database versions, and bioinformatic workflows in full. Cross-study synthesis should first stratify studies according to technical compatibility.

Building on the evidence limitations outlined in Section 3.4, future human OSCC studies should move beyond abundance-based co-detection and directly assess the spatial organization of fungal–bacterial–host interactions. The biological consequences of these interactions are likely to depend on microbial partners, strain variation, spatial organization, nutrient conditions, and host context. Studies of human OSCC should combine microorganism-specific *in situ* methods, such as multiplex fluorescence *in situ* hybridization or RNAscope, with host immune phenotyping in the same lesion. Spatial transcriptomic or metabolomic signals should be considered part of an interaction evidence chain only when the relevant fungi and bacteria are independently localized and appropriate negative controls are included.

### 5.2 Priority research directions

Future longitudinal studies should prioritize standardized repeated fungal measurements and external validation across OPMD subtypes, building on the validation framework outlined in Section 4.2.2. Beyond prognostic association, stronger causal inference will require randomized or otherwise rigorously controlled intervention studies demonstrating that a defined reduction in fungal exposure precedes improvement in clinically relevant oncological outcomes.

Integrating microbial genomics, host transcriptomics, proteomics, and metabolomics can generate testable fungal-bacterial-host mechanisms, but cross-platform correlation alone does not demonstrate interaction (Monteiro et al., 2024; Wicaksono et al., 2025). A stronger design would use paired human lesions to link quantitative fungal burden and strain features with bacterial composition, host-cell states, and metabolites, followed by localization using microorganism-specific spatial methods and perturbation testing in experimentally tractable models. This sequence of evidence would distinguish hypothesis generation, spatial localization, and functional testing.

Candidate microbial interventions, therapeutic targets, and biomarkers require a coherent chain of translational evidence. Evidence for farnesol and repurposed itraconazole derives from OSCC cell or xenograft models; evidence for silver nanoparticles and low-level laser irradiation comes from *in vitro Candida* or infection models; evidence for modulation of fungus-associated immune pathways comes from OSCC cells and tumor-bearing mice; and evidence for *Lactobacillus salivarius* REN comes from a 4NQO-induced rat oral carcinogenesis model. None of these findings is equivalent to established clinical antitumor efficacy in humans (Scheper et al., 2008; Zhang et al., 2013; Seyedmousavi et al., 2014; Hamid et al., 2018; Ban et al., 2020; Wang et al., 2022, 2024; DailyMed, 2026). Before entering clinical studies, each strategy should have a clearly defined molecular or microbial target, achievable local exposure, a pharmacodynamic effect, mucosal and systemic safety, and an assay confirming target engagement. Early human studies should distinguish supportive treatment of candidiasis from tumor modulation and should not use changes in relative microbial abundance alone as evidence of clinical benefit.

In risk prediction, the available evidence provides proof of concept: lesion *Candida* burden was independently associated with malignant transformation in one prospective OPMD cohort, and a study of oral leukoplakia found that an oral microbiome model was associated with the degree of epithelial dysplasia (Galvin et al., 2025; Jiang et al., 2025). The next step is not simply to add microbial variables, but to compare prespecified models against established clinical and histopathological predictors. Studies should report discrimination, calibration, decision-curve analysis or other measures of clinical utility and should evaluate performance in external cohorts. They should also test whether fungal variables provide reproducible incremental information across lesion subtypes, geographic populations, and laboratory workflows.

## 6 Conclusions

Current evidence supports a context-dependent view of *C. albicans* in OSCC. Rather than acting as an independently sufficient carcinogenic driver, *C. albicans* may function as a context-dependent tumor-promoting factor that amplifies OSCC-related processes in susceptible lesion and tumor environments. The evidence reviewed here supports the biological plausibility of several contributory mechanisms, including acetaldehyde production, inflammatory and immune signaling, extracellular-matrix remodeling and invasion, and persistent biofilm-associated colonization. However, much of this evidence derives from cross-sectional human studies and experimental models. The prospective evidence discussed above provides stronger support for temporality between increased fungal burden and malignant transformation, but it does not establish causality or define a clear boundary between passenger colonization and pathological amplification.

Accordingly, *Candida* and host-related measures—including fungal burden, *CaADH1*, SFN, virulence phenotypes, and host-response markers—have research value but have not been validated for diagnosis or clinical risk stratification. Methodological heterogeneity limits direct comparisons across oral mycobiome studies, and fungal–bacterial co-detection in human samples does not itself demonstrate direct cross-kingdom interaction. The therapeutic approaches discussed—including antifungal drug repurposing, biofilm-targeted strategies, combinations with immune-checkpoint blockade, and microbiota modulation—likewise remain preclinical or investigational, with unresolved questions regarding exposure, target engagement, safety, and clinical benefit. Until these requirements are met, *C. albicans* is best regarded as a context-dependent tumor-promoting factor that may amplify OSCC-related processes, rather than an established independent carcinogen, validated biomarker, or therapeutic target.
